# Quality of life among health care workers in Arab countries 2 years after COVID-19 pandemic

**DOI:** 10.3389/fpubh.2022.917128

**Published:** 2022-11-03

**Authors:** Ramy Mohamed Ghazy, Osman Abubakar Fiidow, Fatimah Saed Alabd Abdullah, Iffat Elbarazi, Ismail Ibrahim Ismail, Sulafa Tarek Alqutub, Etwal Bouraad, Esraa Abdellatif Hammouda, Mohamed Mostafa Tahoun, Silmane Mehdad, Rasha Ashmawy, Abdulla Zamzam, Osama Mohamed Elhassan, Qasim Mohamed Al Jahdhami, Hind Bouguerra, Wafaa Kammoun Rebai, Lina Yasin, Esraa Mustafa Jaradat, Yasir Ahmed Mohammed Elhadi, Malik Sallam

**Affiliations:** ^1^Tropical Health Department, High Institute of Public Health, Alexandria University, Alexandria, Egypt; ^2^School of Public Health and Research, Somali National University, Mogadishu, Somalia; ^3^Internal Medicine Department, Alexandria University, Alexandria, Egypt; ^4^Institute of Public Health, College of Medicine & Health Sciences, United Arab Emirates University, Al Ain, United Arab Emirates; ^5^Department of Neurology, Ibn Sina Hospital, Sabah Medical Region, Kuwait City, Kuwait; ^6^Department of Family and Community Medicine, University of Jeddah, Jeddah, Saudi Arabia; ^7^Department of Epidemiology and Population Health Sciences, American University of Beirut, Beirut, Lebanon; ^8^School of Pharmacy, Lebanese International University, Beirut, Lebanon; ^9^Clinical Research Department, El-Raml Pediatric Hospital, Ministry of Health and Population, Alexandria, Egypt; ^10^Department of Epidemiology, High Institute of Public Health, Alexandria University, Alexandria, Egypt; ^11^Research Centre in Genomics of Human Pathologies, Faculty of Science, Mohammed V University in Rabat, Rabat, Morocco; ^12^Department of Clinical Research, Maamoura Chest Hospital, MoHP, Alexandria, Egypt; ^13^Department of Pharmacy, Sidra Medicine, Doha, Qatar; ^14^Quality & Accreditation, Federal Ministry of Health, Khartoum, Sudan; ^15^Ministry of Health, Muscat, Oman; ^16^Faculty of Medicine of Tunis, Tunis, Tunisia; ^17^Laboratory of Biomedical Genomics and Oncogenetics, Institut Pasteur de Tunis, Tunis, Tunisia; ^18^Ministry of Public Health and Population, Sanaa, Yemen; ^19^Ministry of Health, Jerusalem, Palestine; ^20^Department of Public Health, Medical Research Office, Sudanese Medical Research Association, Khartoum, Sudan; ^21^Department of Pathology, Microbiology and Forensic Medicine, School of Medicine, The University of Jordan, Amman, Jordan; ^22^Department of Clinical Laboratories and Forensic Medicine, Jordan University Hospital, Amman, Jordan; ^23^Department of Translational Medicine, Faculty of Medicine, Lund University, Malmö, Sweden

**Keywords:** healthcare providers, health personnel, satisfaction, professional quality of life, COVID-19

## Abstract

**Background:**

Assessment of the quality of life (QoL) among healthcare workers (HCWs) is vital for better healthcare and is an essential indicator for competent health service delivery. Since the coronavirus disease 2019 (COVID-19) pandemic strike, the frontline position of HCWs subjected them to tremendous mental and psychological burden with a high risk of virus acquisition.

**Aim:**

This study evaluated the QoL and its influencing factors among HCWs residing in the Arab countries.

**Methods:**

This was a cross-sectional study using a self-administered online questionnaire based on the World Health Organization QoL-BREF instrument with additional questions related to COVID-19. The study was conducted in three different languages (Arabic, English, and French) across 19 Arab countries between February 22 and March 24, 2022.

**Results:**

A total of 3,170 HCWs were included in the survey. The majority were females (75.3%), aged 18–40 years (76.4%), urban residents (90.4%), married (54.5%), and were living in middle-income countries (72.0%). The mean scores of general health and general QoL were 3.7 ± 1.0 and 3.7 ± 0.9, respectively. Those who attained average physical, psychological, social, and environmental QoL were 40.8, 15.4, 26.2, and 22.3%, respectively. The income per capita and country income affected the mean scores of all QoL domains. Previous COVID-19 infection, having relatives who died of COVID-19, and being vaccinated against COVID-19 significantly affected the mean scores of different domains.

**Conclusion:**

A large proportion of the Arab HCWs evaluated in this study had an overall poor QoL. More attention should be directed to this vulnerable group to ensure their productivity and service provision.

## Introduction

More than 2 years have passed since the World Health Organization (WHO) first announcement of coronavirus disease 2019 (COVID-19) as a worldwide pandemic (1). Indeed, COVID-19 was characterized by variable patterns of spread and mortality rates across different world regions (2, 3). This pandemic impacted all of the 22 members of the Arab League countries. The United Arab Emirates (UAE) was the first country in the Arab League to report the first COVID-19 case on 29 January 2020, while Yemen was the last Arab state to register its first case on 10 April 2020. By the end of May 2020, Egypt was the top-ranked Arab country in the total number of deaths attributed to COVID-19, followed by Algeria, Saudi Arabia, Sudan, and the UAE (4).

Fortunately, there has been a significant decline in COVID-19 severity and mortality after the massive number of vaccines provided (5). However, the pandemic may have long-lasting health effects on healthcare workers (HCWs) (6, 7), including an increased risk of infection (8). The profession as a HCW can be considered as one of the riskiest jobs, since health professionals are continually exposed to a wide range of occupational health and safety concerns. The risks vary from biological exposure to disease-causing organisms or exposure to chemicals (9). The COVID-19 added an extra burden on the fragile health sector, particularly in low-income countries. The WHO estimates that between 80,000 and 1,80,000 HCWs could have died due to COVID-19 during the period from January 2020 to May 2021, converging to a medium estimate of 1,15,500 deaths. It is noteworthy that the Eastern Mediterranean Region (EMR) was ranked 3rd in terms of the number of deaths among HCWs, being preceded by Europe and the Americas (10).

The quality of life (QoL) is a broad concept that delineates “an individual's perception of their position in life” (11). This subjective evaluation comes in context of the culture and value systems in which they live and with their goals, expectations, standards, and concerns (12). Given the high number of deaths among HCWs, the COVID-19 pandemic has been perceived to significantly influence the QoL of HCWs. Indeed, hospital settings are characterized by a high level of work-related stress, which is a known factor that is associated with an increased risk of low QoL. For example, a recent study in Malaysia that was reported in 2021 showed that HCWs had lower QoL scores in social relationship than the normal population. Moreover, COVID-19 related stressors such as annual leave freeze, loss of daily routine, frequent exposure to COVID-19 cases, and psychological distress were considered as predictors of low QoL scores among HCWs (13). Recent reports among HCWs in Egypt and Saudi Arabia showed that the participants experienced depression, anxiety, stress, and inadequate sleep during the COVID-19 pandemic, impacting their QoL (14). Moreover, a recent study from Saudi Arabia reported that the emergence of Omicron variant of severe acute respiratory distress coronavirus 2 (SARS-CoV-2) was associated with increased stress and uncertainty as well as reduced resilient coping scores among a group of HCWs in the country (15).

Despite the noticeable growth in literature addressing the impact of COVID-19 pandemic on frontline workers including health professionals, the QoL among HCWs is a research topic that requires further investigation, especially in the Arab countries. Several challenges may impact HCWs' health QoL and these challenges vary across different Arab countries based on distinctive socio-cultural, environmental, economic, and political contexts. More studies are needed to depict these challenges at the country and regional levels. To face the challenges of healthcare delivery systems and to ensure the quality of care and client satisfaction with the care received, it is critical to assess HCWs-QoL and the role of its associated factors (16). The current study aimed to estimate the QoL scores and to investigate its determinants among HCWs living and working in Arab countries 2 years from the declaration of COVID-19 as a pandemic using the short version of the World Health Organization QoL (WHOQOL-BREF) instrument.

## Methodology

A cross-sectional online open survey was conducted using the convenience sampling method to recruit HCWs (physicians, dentists, pharmacists, nurses, paramedical, and administrative staff in healthcare settings). Eligible participants were those aged 18 years or older and living in one of the Arab countries. Participants from Algeria, Djibouti, and Comoros were excluded due to low response rate from these countries. The valid short form of the WHO-QOL-BREF questionnaire (17), was circulated *via* different social media platforms (Facebook, WhatsApp, and Twitter) and through E-mail in three languages (Arabic, English, and French). A focal point (researcher) from each country was assigned to collect data. The electronic form of the questionnaire was tested technically and piloted in three countries (Egypt, Jordan, and Saudi Arabia) to assess the response rate of 75% and time required to fill in the questionnaire. The questionnaire was adjusted accordingly. All the responses collected during the pilot testing were excluded from the final analysis. The survey was collected between February 22, 2022 and March 24, 2022. We followed the Checklist for Reporting Results of Internet E-Surveys (CHERRIES). [Supplementary material 1 (18)].

### Sampling

A minimum required sample was specified to be 3,072 based on the following sampling formula: the minimum required sample size (*N*) = ([Z^2^ × P × (1–P)/(E)^2^] × Deff × income level estimate)/expected response rate. *N* = (1.962^2^ × 0.5 (1–0.5)/(0.05)^2^ × 1.5 × 4)/0.75 (19).

### Data collection

The final survey consisted of three sections; the first section included questions on sociodemographic data. The second section comprised questions on the history of chronic diseases, profession, history of COVID-19 infection, vaccination status [fully vaccinated (got all COVID-19 primary series dose/doses), partially vaccinated (did not get all COVID-19 primary series doses), not vaccinated (did not receive any doses of vaccine), or received booster dose (received booster dose of the bivalent mRNA vaccines at least 2 months after the primary series doses)], and history of deaths among relatives due to COVID-19 infection. The third section included the validated WHOQOL-BREF instrument (17). The questionnaire was administered in English, Arabic, and French languages. A team of researchers reviewed and finalized language editing with excellent Arabic, English and French language skills (RG, EA, and EBR) (20, 21). The WHOQOL-BREF consists of 26 items, two items for evaluating general QoL and general health, and 24 items for assessing QoL in four domains, namely physical (seven items), psychological (six items), social relationship (three items), and environmental domain (eight items). The tool follows a scoring system, where each question is rated on a 5-point Likert scale, ranging from 1 (very poor/very dissatisfied/none/ never) to 5 (very good/very satisfied/extremely/always). Then the scores of all four domains were summed and scaled positively, transformed to a 0–100 scale with higher scores indicating better QoL. The estimated acceptable values of the QoL domains in the general population are as follows: physical health QoL = 73.5 ± 18.1, psychological QoL = 70.6 ± 14.0, social relationship QoL = 71.5 ± 18.2, and environmental QoL = 75.1 ± 13.0. Respondents whose scores were above these thresholds were classified as having good quality, while those with scores below the thresholds were classified as having poor quality (22).

### Ethical consideration

The study was approved by the ethical committee of the Faculty of Medicine, Alexandria University, Egypt (IRB: 0305505). The research was conducted in compliance with Declaration of Helsinki. The study information was provided in the front page of an open survey, voluntary participation was considered as an informed consent to all participants.

### Statistical analysis

Descriptive statistics were calculated for the socio-demographics factors, COVID-19 information, and QoL profiles. Categorical variables were presented as percentages and continuous variables as means ± standard deviation (SD). Assumptions of normal distribution were explored with the Kolmogorov-Smirnov test and visual inspections of the histograms. We used the Pearson correlation coefficients, as appropriate, to examine the relationships between different domains. Independent *t*-test, ANOVA, and MANOVA with Bonferroni's method to adjust for the number of comparisons, were used to investigate differences between QoL domains. Multiple linear regression analyzes were conducted to identify the determinants of different domains of QoL. Tolerance and variance inflation factor (VIF) were utilized to verify multicollinearity. Tolerance levels <0.10 and VIF values >10 often indicate problems with multicollinearity (23). Different domains of QoL were compared across countries based on their income per capita. Countries were categorized according to the World Bank Classification into (low-income, lower-middle income, higher-middle income, and high-income) (24). The data were analyzed using the statistical software IBM for Windows (SPSS Inc., Chicago, Illinois version 26) and STATA 14.2.

## Results

### Study population characteristics

**Figure 1:**
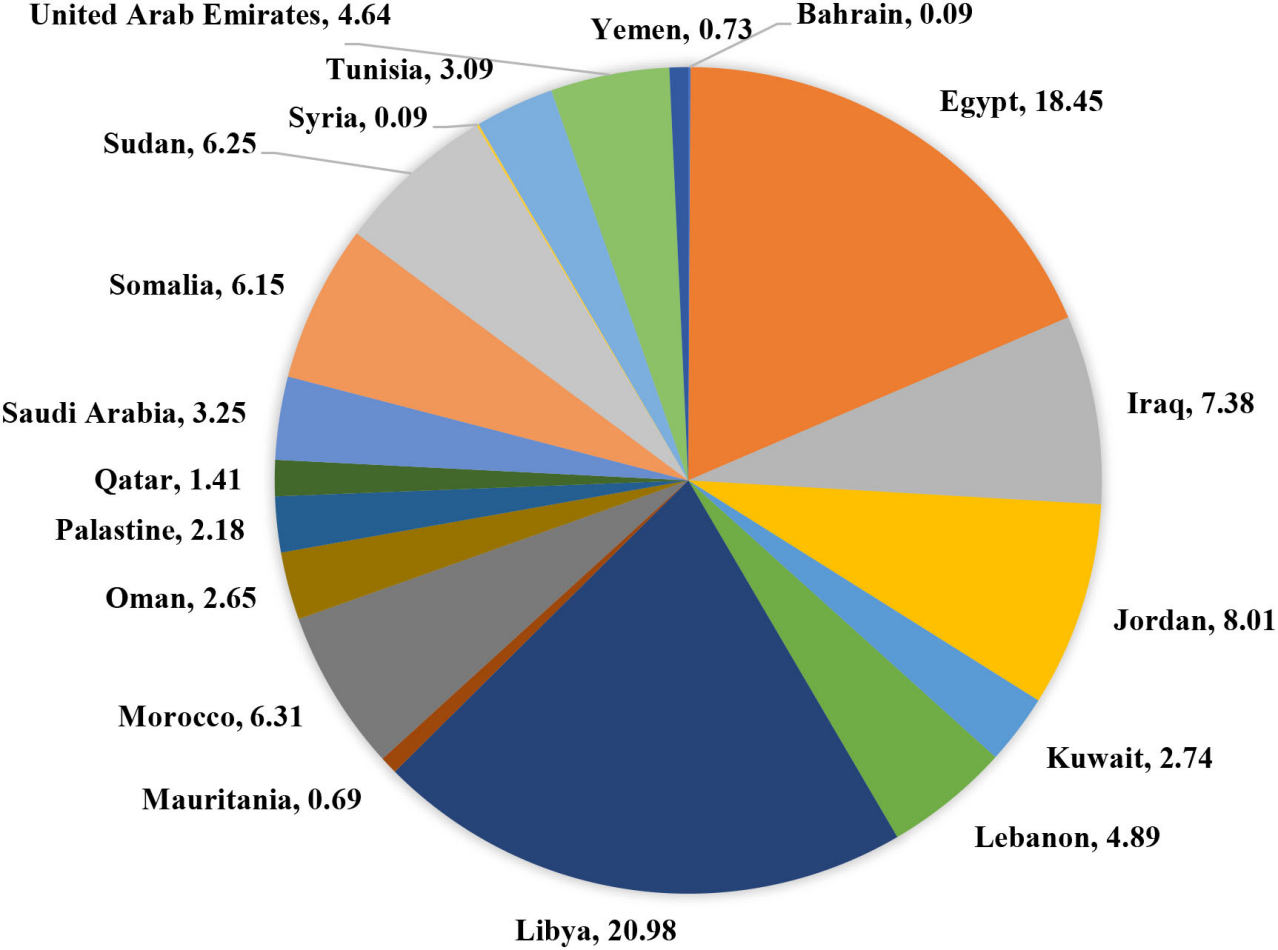
Country of residence of the respondents.

A total of 3,170 HCWs from 19 Arab countries were included in this survey. The proportion of participants from each country is shown in (Figure 1). Regarding languages used by the study respondents, 75.3% responded in Arabic, 18.6% in English, and 6.1% in French. Among the surveyed HCWs, 75.3% were females, 76.4% aged 18–40 years, 54.5% were married, 90.4% were living in urban areas, 72.0% were from middle-income countries, 21.7 % reported not enough income, 70.0% had crowding index of <2, 54.5% had a university degree, 55.9% were working for the governmental sector, 48.3% were physicians. Regarding the health characteristics, 40.7% had work experience of <5 years, 22.5% had chronic diseases, 52.5% reported a previous SARS-CoV-2 infection, 51.6% were fully vaccinated against COVID-19, and 37.4% had relatives who died due to COVID-19 (Table 1).

**Table 1:** Sociodemographic and health related characteristics of the study participants.

**Variables (*****n*** = **3,170)**		***n* (%)**
Language	Arabic	2,389 (75.2)
	English	591 (18.6)
	French	194 (6.1)
Sex	Female	2,385 (75.2)
	Male	784 (24.8)
Age	18–30	1,305 (41.2)
	31–40	1,117 (35.2)
	41–50	443 (14.0)
	51–60	230 (7.2)
	>60	6 (0.2)
Marital status	Divorced	89 (2.8)
	Married	1,729 (54.5)
	Single	1,321 (41.7)
	Widow	31 (1.0)
Residence	Rural/remote area	305 (9.6)
	Urban	2,865 (90.4)
Country income level	Low-income	419 (13.2)
	Lower-middle income	974 (30.7)
	Higher-middle income	1,308 (41.3)
	High income	469 (14.8)
Income per capita	Enough	1,717 (54.2)
	Enough and save	765 (24.1)
	Not enough and borrowing large sums	100 (3.2)
	Not enough and borrowing small amounts	485 (15.3)
	Not enough, and he/she is in debt, and he/she cannot fulfil the debt	103 (3.2)
Crowding index	<2	2,220 (70.0)
	From 2 to 4	865 (27.3)
	Above 4	85 (2.7)
Educational level	Preparatory	6 (0.2)
	Primary	5 (0.2)
	Secondary	82 (2.6)
	University graduate	1,725 (54.4)
	Post-graduate	1,352 (42.6)
Working sector	Governmental sector	1,771 (55.9)
	Not working (including trainees, volunteers, retired)	663 (20.9)
	Private sector	736 (23.2)
Occupation	Co-worker in health sector	243 (7.7)
	Nurse	341 (10.7)
	Pharmacist	672 (21.2)
	Physician (include dentist)	1,529 (48.2)
	Physiotherapist	50 (1.6)
	Technician (laboratory, radiology)	335 (10.6)
Years of work experience	≥ 15 years	710 (22.4)
	5–10 years	1,171 (36.9)
	<5 years	1,289 (40.7)
Chronic diseases		714 (22.5)
Confirmed infection with COVID-19		1,667 (52.5)
COVID-19 vaccination	Fully vaccinated	1,633 (52.5)
	Not vaccinated	663 (20.9)
	Partially vaccinated	331 (10.4)
	Received the booster dose	573 (18.1)
Have relative died due to COVID-19		1,186 (37.4)

### The mean scores of the QoL domains of the studied health care workers

The mean score of all domains of QoL among HCWs was 59.4 ± 13.0, with 53.5 ± 23.0 for the social domain, 55.9 ± 17.9 for the environmental domain, and 60.3 ± 12.7 for the psychological domain. The highest mean score was for the physical domain, 68.0 ± 15.7. The mean score of general health and general QoL were 3.7 ± 1.0 and 3.7 ± 0.9, respectively. Interestingly, participants who attained normal physical, psychological, social, and environmental domains of QoL were 40.8%, 15.4%, 26.2%, and 22.3%, respectively (Table 2).

**Table 2:** Summary of the quality-of-life scores from WHOQoL-BREF domains.

**Item**	**Min**	**Max**	**Mean ±SD**	**Cut-off point**	**Normal *n* (%)**
Physical	10.7	100.0	68.0 ± 15.7	73.5	1,294 (40.8)
Psychological	12.5	100.0	60.3 ± 12.7	70.6	489 (15.4)
Social relation	8.3	100.0	53.5 ± 23.0	71.5	832 (26.2)
Environment	0.0	100.0	55.9 ± 17.9	75.1	707 (22.3)
General health	1.0	5.0	3.7 ± 1.0	–	1,921 (60.6)
General QoL	1.0	5.0	3.7 ± 0.9	–	1,940 (61.2)
Total score	6.7	93.3	59.4 ± 13.0	59.4	1,657 (52.3)

The mean score of QoL of all domains was higher in co-workers (61.11 ± 13.84) and nurses (60.28 ± 13.87) compared to the scores of pharmacists and physiotherapist (58.06 ± 12.42), physicians (59.76 ± 12.76), and medical technicians (58.66 ± 13.18, Table 3).

**Table 3:** Quality of life of different categories of health care workers.

**Profession**	**Proportion (%)**	**Proportion 95% CI**	**Mean (SD) QoL for all domains**
Co-workers	0.077 (7.7)	0.068–0.086	61.11 ± 13.84
Nurses	0.108 (10.8)	0.097–0.118	60.28 ± 13.87
Pharmacists	0.212 (21.2)	0.198–0.226	58.06 ± 12.42
Physicians	0.482 (48.2)	0.465–0.499	59.76 ± 12.76
Physiotherapist	0.016 (1.6)	0.012–0.021	58.06 ± 14.74
Technicians	0.106 10.6	0.095–0.117	58.66 ± 13.18

### Correlation between the scores of different domains of QoL

**Figure 2:**
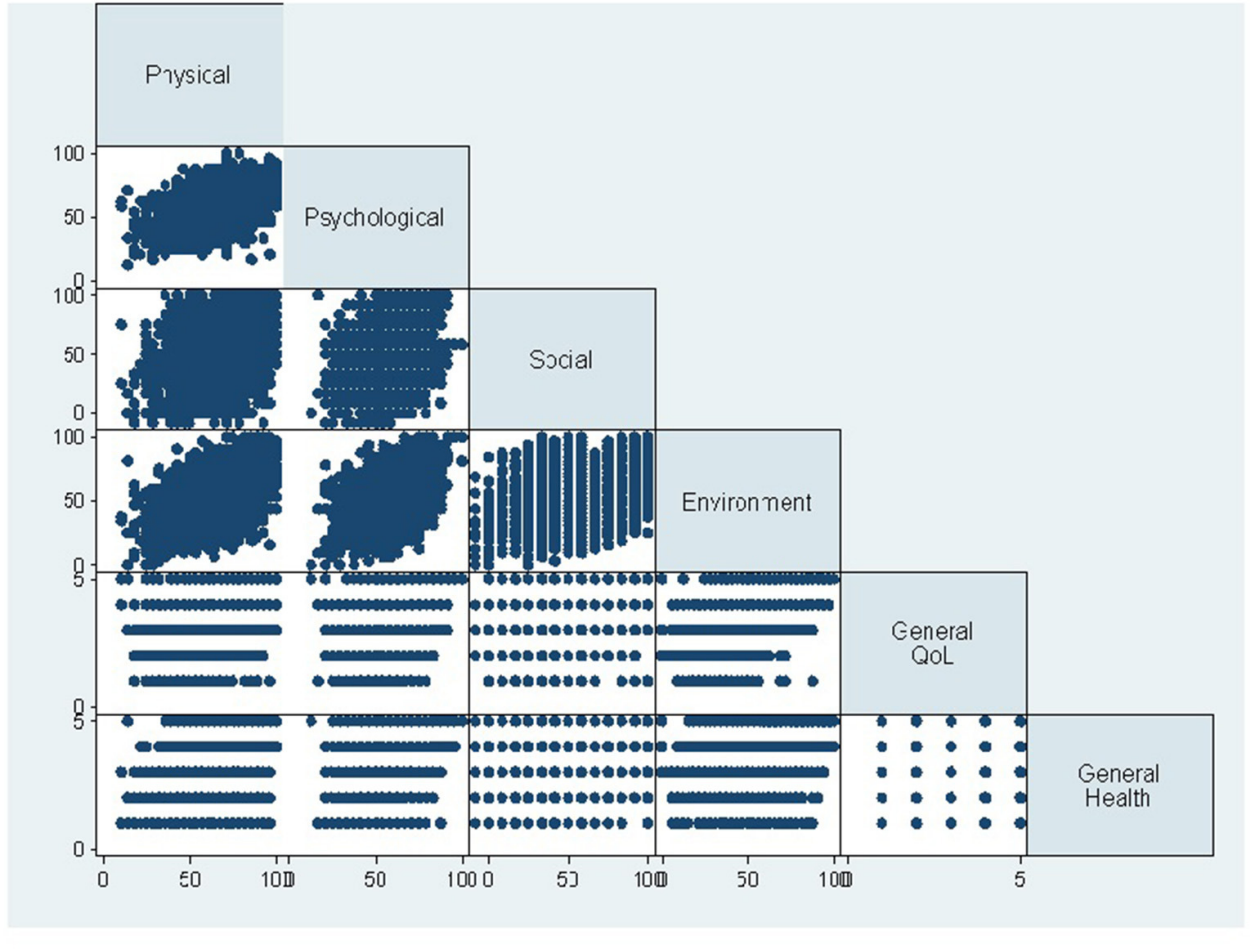
The correlation between different domains of quality of life (QoL), general health, and general QoL.

The different domains of QoL showed positive correlation with each other. Physical domain showed statistically significant correlation with psychological domain (*r* = 0.52, *p* < 0.001), social domain (*r* = 0.29, *p* < 0.001), environmental domain (*r* = 0.53, *p* < 0.001), general QoL (*r* = 0.35, *p* < 0.001), and general health (*r* = 0.46, *p* < 0.001). Psychological domain was significantly correlated with social domain (*r* = 0.35, *p* < 0.001), and environmental domain (*r* = 0.53, *p* < 0.001), general QoL (*r* = 0.41, *p* < 0.001), and general health (*r* = 0.36, *p* < 0.001). Social domain was significantly correlated with environmental domain (*r* = 0.35, *p* < 0.001), general QoL (*r* = 0.23, *p* < 0.001), and general health (*r* = 0.21, *p* < 0.001). Environmental domain showed positive correlation with general QoL (*r* = 0.58, *p* < 0.001), and general health (*r* = 0.38, *p* < 0.001). General QoL showed positive correlation with general health (*r* = 0.38, *p* < 0.001, Figure 2).

### Sociodemographic factors and quality of life among health care workers

The participating HCWs who were 40 years or older had higher mean scores of psychological domains (60.0 ± 12.8 vs 61.2 ± 12.3), with lower social domain mean score (51.0 ± 23.1 vs 61.6 ± 20.9), and environmental domain (55.4 ± 17.8 vs 57.7 ± 18.1) (*p* < 0.05) than their counterpart aged below 40 years. Male HCWs had significantly lower mean scores of physical and social domains of QoL than females (67.3 ± 15.5 vs 70.2 ± 16.1) and (55.7 ± 17.6 vs 56.7 ± 18.7), respectively. Compared to HCWs living in rural areas, those living in urban settings had significantly higher scores of all domains [physical (65.7 ± 16.0 vs 58.3 ± 13.7, psychological (50.7 ± 22.9 vs 51.7 ± 17.8), social (68.3 ± 15.7 vs 60.5 ± 12.5), and environmental (53.8 ± 23.0 vs 56.4 ± 17.8). Except for the physical domain, the participants from high-income countries had higher scores in all domains. In addition, married HCWs mean scores were higher in terms of their social health (65.0 ± 19.3 vs 39.7 ± 19.3). The participating HCWs with post-graduate degrees had higher scores in all QoL domains compared to other levels of education. The participants working in the private sector had higher physical and environmental health scores. Public sector HCWs had higher scores in terms of social relations. Furthermore, physicians had higher mean scores of psychological and environmental domains upon comparison to other HCWs who were grouped together as non-physicians (60.8 ± 12.3 vs 59.8 ± 13.0) and (57.0 ± 17.7 vs 54.9 ± 18.0), respectively. The participating HCWs who reported work experience of more than 10 years had higher mean environmental and social scores (Table 4).

**Table 4:** Sociodemographic factors and quality of life.

**Variable**	**Physical domain**	**Psychological domain**	**Social relationship**	**Environment**
	**Mean ± SD**	**Mean ± SD**	**Mean ± SD**	**Mean ± SD**
**Age**				
<40 years	68.2 ± 15.8	60.0 ± 12.8	51.0 ± 23.1	55.4 ± 17.8
≥40 years	67.4 ± 15.5	61.2 ± 12.3	61.6 ± 20.9	57.7 ± 18.1
*p^*a*^*	0.22	**0.02**	**0.001**	**0.002**
**Gender**				
Male	67.3 ± 15.5	60.4 ± 12.5	52.15 ± 23.0	55.7 ± 17.6
Female	70.2 ± 16.1	59.9 ± 13.3	57.56 ± 22.6	56.7 ± 18.7
*p^*a*^*	**0.001**	0.377	**0.001**	0.181
**Residence**				
Rural	65.7 ± 16.0	58.3 ± 13.7	50.7 ± 22.9	51.7 ± 17.8
Urban	68.3 ± 15.7	60.5 ± 12.5	53.8 ± 23.0	56.4 ± 17.8
*p^*a*^*	**0.006**	**0.004**	**0.024**	**0.001**
**Chronic diseases**				
Yes	68.0 ± 15.68	60.8 ± 12.4	54.0 ± 23.2	56.7 ± 18.0
No	68.0 ± 15.7	60.1 ± 12.8	53.4 ± 22.9	56.0 ± 17.8
*p^*a*^*	0.986	0.206	0.534	0.164
**Country income level**				
Low-income	70.9 ± 17.0	60.2 ± 13.7	53.1 ± 23.5	53.7 ± 19.2
Lower-middle	65.5 ± 15.3	59.0 ± 12.1	55.4 ± 22.1	53.9 ± 16.5
Higher-middle	68.7 ± 15.7	60.9 ± 13.0	49.6 ± 23.2	54.1 ± 17.8
High	68.6 ± 14.5	61.0 ± 11.7	60.9 ± 21.4	67.1 ± 15.6
*p^*b*^*	**0.002**	**0.002**	**0.001**	**0.001**
**Marital status**				
Married	67.6 ± 15.2	60.9 ± 12.2	65.0 ± 19.3	55.9 ± 17.2
Single	68.5 ± 16.3	60.0 ± 13.3	39.7 ± 19.3	56.0 ± 18.9
*p^*a*^*	0.1160	0.258	**0.001**	0.865
**Education**				
Primary/secondary	62.9 ± 16.7	57.3 ± 15.9	53.4 ± 23.2	51.8 ± 19.3
University level	68.1 ± 16.0	60.10 ± 12.8	50.7 ± 23.6	55.6 ± 18.1
Post-graduate level	68.3 ± 15.2	60.64 ± 12.2	57.1 ± 21.7	56.7 ± 17.4
*p^*b*^*	**0.006**	**0.04**	**0.001**	**0.018**
**Working sector**				
Government sector	67.4 ± 15.0	60.3 ± 12.1	57.7 ± 21.8	55.1 ± 17.1
Not working (including trainees, volunteers, and retired)	67.8 ± 17.6	59.6 ± 14.0	41.3 ± 21.5	56.2 ± 19.7
Private sector	69.7 ± 15.5	60.5 ± 12.8	53.9 ± 23.2	57.7 ± 18.1
*p^*b*^*	**0.004**	0.332	**0.001**	**0.004**
**Health profession**				
Physicians	68.6 ± 15.5	60.8 ± 12.3	52.8 ± 22.7	57.0 ± 17.7
Non-physician	67.5 ± 15.8	59.8 ± 13.0	54.2 ± 23.3	54.9 ± 18.0
*p^*a*^*	0.065	**0.028**	0.085	**0.001**
**Experience**				
≥10 years	67.6 ± 15.7	61.2 ± 12.5	61.9 ± 20.8	58.0 ± 17.6
5–10 years	67.6 ± 15.2	59.9 ± 12.0	57.5 ± 22.0	55.3 ± 17.4
<5 years	68.6 ± 16.1	60.1 ± 13.3	45.2 ± 22.3	55.4 ± 18.3
*p^*b*^*	0.243	0.076	**0.001**	**0.002**

p^a^ = T-test p-Value.

p^b^ = ANOVA p-Value. The bold values indicates significant.

For the country income level, there was a significant difference between the scores for the lower-middle vs low-income countries within the physical domain (*p* < 0.001); higher-middle vs lower-middle income countries (*p* < 0.001); high vs lower-middle income countries (*p* = 0.003). Within the psychological domain, there was a significant difference between higher-middle vs lower- middle income countries participants' scores (*p* = 0.002); high vs lower-middle income countries participants' scores (*p* = 0.030). Within the social relationships there was significant difference (*p* < 0.001) between the scores for all country income levels except between the lower-middle vs low-income countries. As for the environment domain there was a statistical difference between high vs low (*p* < 0.001); high vs lower-middle (*p* < 0.001) and high vs higher-middle income countries (*p* < 0.001). For education, within the physical domain, there was no significant difference between university level vs primary and secondary education mean score (*p* = 0.06), while there was statistically significant difference between post-graduate vs primary and secondary education mean score (*p* = 0.040). Within the psychological domain, there was only a significant difference (*p* = 0.040) between post-graduate vs primary and secondary education. For the social relationship there was only post-graduate vs university level (*p* < 0.001). As for the environment domain, the only statistical difference was shown between university level vs primary and secondary level (*p* = 0.030). For the work sector, within the physical domain, there was a significant difference between the private vs government sector (*p* = 0.030). Within the psychological domain, there was no statistical difference between any levels. For the social relationship there was a statistically significant difference between all work sector groups (*p* < 0.001). With respect to the working experience, there was a significant difference between the social relationships mean score between all groups (*p* < 0.001). As for the environment score, there was a statistically significant difference between all groups (*p* < 0.010) except between <5 years and between 5–10 years (Table 5).

**Table 5:** *Post-hoc* analysis for the one-way ANOVA to differentiate among different groups across the domains of QoL.

**Variables**	**Physical domain**		**Psychological domain**		**Social relationship**		**Environment**	
	**Mean difference**	***p*-Value**	**Mean difference**	***p*-Value**	**Mean difference**	***p*-Value**	**Mean difference**	***p*-Value**
**Country income level**								
Lower-middle vs low	−5.38	<0.001	−0.28	0.393	0.28	0.298	0.23	0.995
Higher-middle vs low	−2.17	0.063	0.18	0.705	−0.42	0.03	0.453	0.996
High vs low	−2.36	0.108	0.19	0.784	0.93	<0.001	13.37	<0.001
Higher-middle vs lower-middle	3.2	<0.001	0.46	0.02	−0.7	<0.001	0.217	0.991
High vs lower-middle	3.02	0.03	0.47	0.03	0.66	<0.001	13.14	<0.001
High vs higher-middle	−0.194	0.996	0.08	1	1.37	<0.001	12.92	<0.001
**Education**								
University vs primary/secondary	5.14	0.06	0.66	0.102	−0.32	0.504	3.77	0.116
Post-graduate vs primary/secondary	5.33	0.004	0.789	0.041	0.44	0.295	4.89	0.029
University vs post-graduate	0.19	0.936	0.128	0.477	0.76	<0.001	1.113	0.2
**Working sector**								
Not working vs government	0.346	0.878	−0.19	0.359	−1.98	<0.001	1.06	0.39
Private vs not working	2.25	0.03	0.03	0.974	−0.47	<0.001	2.6	0.003
Not working vs private	1.911	0.059	0.218	0.372	1.51	<0.001	1.54	0.241
**Experience**								
5–10 years vs ≥10 years	−0.014	1	−0.32	0.072	−0.52	<0.001	−2.68	0.005
≤10 years vs ≥10 years	0.944	0.402	−0.26	0.158	−2	<0.001	−2.63	0.004
≤10 years vs 5 years	0.96	0.285	0.057	0.887	−1.47	<0.001	−0.43	0.998

### COVID-19 infection and quality of life of healthcare workers

The participating HCWs who were previously diagnosed with COVID-19 infection had a significantly higher mean physical (69.6 ± 15.1 vs 66.9 ± 16.1) and psychological (60.7 ± 12.5 vs 59.8 ± 12.8) scores of QoL when compared to those who have not been infected. Similarly, fully vaccinated HCWs had higher environmental health scores compared with those who are not fully vaccinated (55.5 ± 17.7 vs 52.5 ± 18.00). Furthermore, HCWs who reported no COVID-19 related death among relatives had higher mean scores of physical (69.29 ± 15.54 vs 65.87 ± 15.73) and environmental domains (57.3 ± 17.8 vs 53.6 ± 17.8) (Table 6).

**Table 6:** COVID19 factors and quality of life.

**Variable**	**Physical domain**	**Psychological domain**	**Social relationship**	**Environment**
	**Mean ± SD**	**Mean ± SD**	**Mean ± SD**	**Mean ± SD**
**Pervious COVID 19 infections**				
No	69.61 ± 15.05	60.73 ± 12.51	52.95 ± 23.35	56.19 ± 17.85
Yes	66.58 ± 16.12	59.82 ± 12.80	53.96 ± 22.67	55.68 ± 17.92
*p*-Value	**0.001**	**0.044**	0.217	0.412
**COVID 19 vaccination**				
Full vaccinated	67.72 ± 15.87	60.36 ± 12.51	53.11 ± 23.32	55.48 ± 17.73
Not vaccinated	69.07 ± 16.03	60.05 ± 13.61	52.86 ± 23.03	52.49 ± 18.01
*p*-Value	0.07	0.65	0.814	**0.001**
**Relative died due to COVID 19**				
No	69.29 ± 15.54	60.58 ± 12.66	53.82 ± 23.04	57.31 ± 17.82
Yes	65.87 ± 15.73	59.69 ± 12.67	52.93 ± 22.92	53.60 ± 17.75
*p*-Value	**0.001**	0.053	0.293	**0.001**

Independent T-test. SD, standard deviation. The bold values indicates significant.

### Impact of household income per capita on quality of life

**Figure 3:**
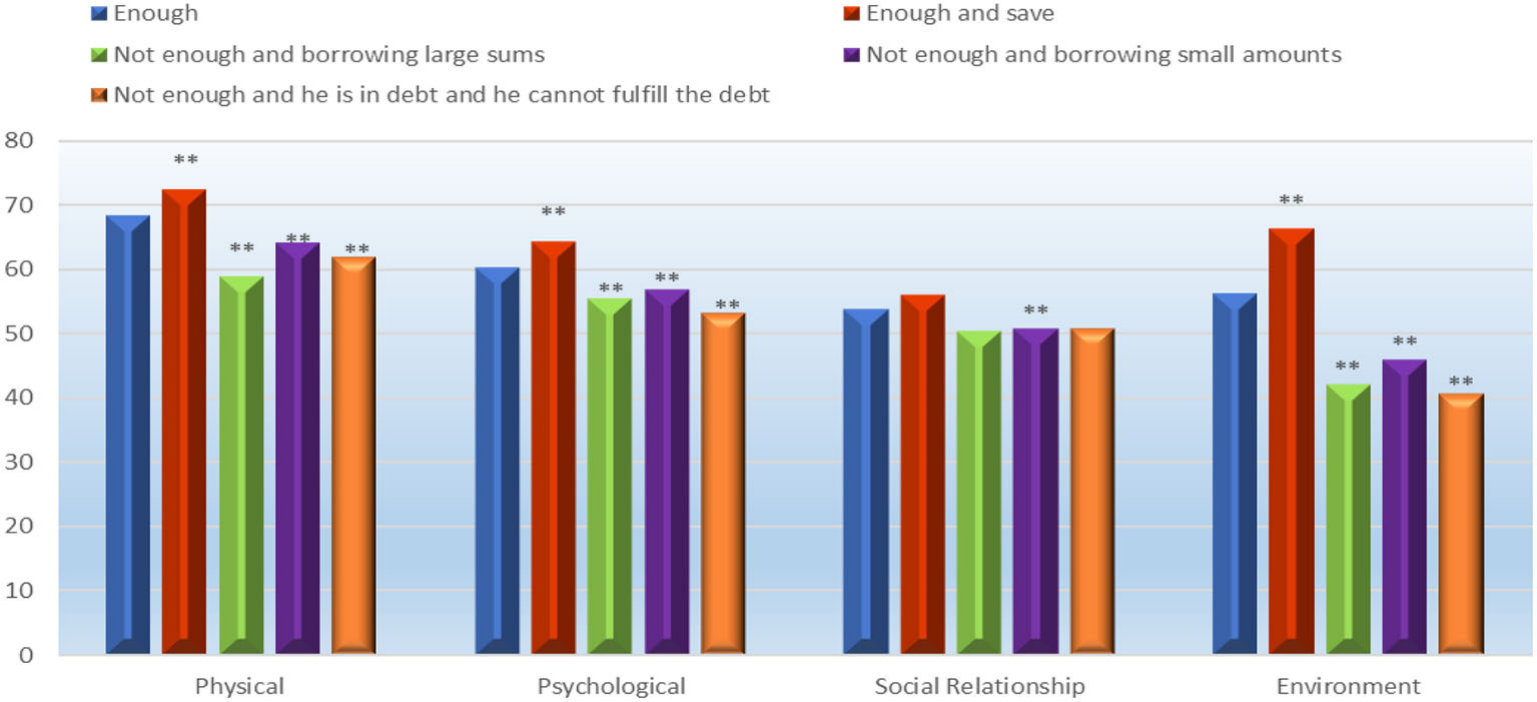
One-way multivariate analysis of variance (MANOVA) in testing the effect of income on QoL different domains. Bonferroni's method adjustments for the number of comparisons were applied. ^**^p < 0.001.

After controlling the covariates, multivariate analyzes of covariance were used to determine the impact of household income per capita on QoL domains. Pillai's trace statistic showed a significant effect for the household income per capita on physical, psychological, social, and environmental domains after controlling the demographic factors (age, gender, education, and marital status); V = 0.18, F = 37.7, *p* < 0.001, ηp^2^ = 0.05, which can be interpreted as a small to medium effect. The mean of QoL domains indicated that participants having enough income and saving had higher QoL (physical, psychological, social, and environmental health) (Figure 3).

### Determinants of the QoL of health care workers

Multilinear regression analysis revealed that age and years of experience did not affect QoL in any domain. On the other hand, insufficient income per capita significantly decreased QoL, while the male gender had higher QoL in physical and social domains. Urban residence significantly increased physical, psychological, and environmental QoL. In addition, being single, working in a governmental sector, and being a physician caused 24.2, 4.9, and 2.8 decreases in social domain scores. For COVID-19 variables, the physical domain score decreased significantly if the subject had a previous COVID infection (β = −2.6 and β = −1.03). Having a relative who passed because of COVID-19 decreased the physical and the environmental scores (β = 3.08 and β = −2.9, respectively). Environmental score decreased significantly if the respondent was not vaccinated (β = −2.5) (Table 7).

**Table 7:** Predictors of different domains of QoL.

**Variable**	**Physical domain**	**Psychological domain**	**Social domain**	**Environment domain**
	**β [95%CI]**	**β [95%CI]**	**β [95%CI]**	**β [95%CI]**
Intercept	^*^68.2 [65.0, 71.4]	^*^60.2 [57.6, 62.8]	^*^70.4 [66.4, 74.4]	^*^64.5 [61.1, 67.8]
**Age**				
<40 years	Ref	Ref	Ref	Ref
≥40 years	1.01 [−2.9, 0.9]	0.7 [−0.8, 2.3]	−0.9 [−3.3, 1.5]	0.13 [−1.8, 2.1]
**Gender**				
Male	^*^3.15 [1.86, 4.44]	−0.3 [−1.4, 0.7]	^*^2.7 [1.1, 4.4]	−0.6 [−1.9, 0.8]
Female	Ref	Ref	Ref	Ref
**Residence**				
Rural	Ref	Ref	Ref	Ref
Urban	^*^2.19 [0.39, 3.98]	^*^1.5 [0.05, 3.0]	0.2 [−2.1, 2.5]	^*^2.4 [0.5, 4.2]
**Country income level**				
Low	^*^4.1 [1.9, 6.3]	1.7 [−0.1, 3.5]	2.2 [−0.6, 4.9]	^*^−7.8 [−10.1, −5.5]
Lower-middle	−1.4 [−3.18, 0.28]	−1.1 [−2.5, 0.4]	^*^−3.9 [−6.1, −1.7]	^*^−10.4 [−12.2, −8.6]
Higher-middle	^*^2.7 [0.97, 4.47]	^*^1.6 [0.2, 3.1]	^*^−2.4 [−4.6, −0.2]	^*^−9.9 [−11.8, −8.2]
High	Ref	Ref	Ref	Ref
**Marital status**				
Married	Ref	Ref	Ref	Ref
Single	0.04 [−1.15, 1.36]	−0.4 [−1.4, 0.6]	^*^−24.2 [−25.7, −22.5]	0.7 [−0.6, 2.02]
**Chronic disease**				
No	Ref	Ref	Ref	Ref
Yes	0.02 [−1.23, 1.28]	0.5 [−0.5, 1.6]	−0.001 [−1.6, 1.6]	0.3 [−1.02, 1.6]
**Education**				
Primary/secondary University level	^*^−4.1 [−7.3, −0.76]	−1.8 [−4.4, 0.9]	1.2 [−2.9, 5.3]	−1.6 [−5.02, 1.8]
Post-graduate level	−0.69 [−1.88, 0.63]	^*^−0.01 [−1.04, 1.02]	−0.14 [−1.7, 1.4]	0.5 [−0.8, 1.8]
	Ref	Ref	Ref	Ref
**Working sector**				
Government sector	Ref	Ref	Ref	Ref
Not working	−1.26 [−2.99, 0.3]	^*^−1.5 [−2.8, −0.13]	^*^−4.9 [−7.03, −2.8]	1.4 [−0.3, 3.2]
Private sector	0.4 [−1.01, 1.8]	−0.3 [−1.5, 0.8]	−0.7 [−2.5, 1.06]	1.3 [−0.1, 2.8]
**Health profession**				
Physicians	−0.28 [−1.55, 0.76]	−0.3 [−1.3, 0.6]	^*^−2.8 [−4.3, −1.4]	0.7 [−0.5, 1.9]
Non-physician	Ref	Ref	Ref	Ref
**Experience**				
≥10 years	Ref	Ref	Ref	Ref
5-10 years	−0.93 [−2.87, 1.03]	−0.9 [−2.5, 0.7]	−1.6 [−4.1, 0.7]	−1.8 [−3.8, 0.3]
<5 years	−0.36 [−2.59, 1.96]	−0.3 [−2.2, 1.5]	−1.4 [−4.2, 1.4]	−1.6 [−3.9, 0.8]
**Income per capita**				
Enough	Ref	Ref	Ref	Ref
Enough save	^*^4.3 [3.02, 5.6]	^*^4.1 [3.03, 5.2]	^*^2.3 [0.7, 4.01]	^*^8.6 [7.3, 10.01]
Not enough +	^*^−4.45 [−5.9, −2.9]	^*^−3.4 [−4.7, −2.2]	^*^−3.6 [−5.5, −1.6]	^*^−9.8 [−11.4, −8.2]
Not enough ++	^*^−9.4 [−12.5, −6.3]	^*^−4.8 [−7.4, −2.3]	−3.6 [−7.4, 0.3]	^*^−13.3 [−16.5, −1.01]
Not enough +++	^*^−6.9 [−9.9, −3.9]	^*^−6.8 [−9.3, −4.4]	−3.5 [−7.3, 0.2]	^*^−15.02 [−18.2, −11.9]
**Pervious COVID-19 infections**				
No	Ref	Ref	Ref	Ref
Yes	^*^−2.62 [−3.7, −1.6]	^*^−1.03 [−1.9, −0.15]	−0.03 [−1.4, 1.3]	−0.7 [−1.8, 0.4]
**Relative died due to COVID-19**				
No	Ref	Ref	Ref	Ref
Yes	^*^−3.08 [−4.2, −1.9]	−0.7 [−1.6, 0.16]	−0.9 [−2.4, 0.4]	^*^−2.9 [−4.02, −1.7]
**COVID-19 vaccination**				
Fully vaccinated	Ref	Ref	Ref	Ref
Partially vaccinated	−0.14 [−1.9, −1.6]	−0.7 [−1.7, 0.7]	^*^−2.8 [−5.1, −0.6]	^*^−2.1 [−3.9, −0.24]
Not vaccinated	0.11 [−1.4, 1.6]	−0.5 [−2.1, 0.8]	0.19 [−1.6, 2.05]	^*^−2.5 [−4.1, −1.01]
**Model statistics**				
F-statistics (df) *p*-value	13.83 (3,169)	9.55 (3,169)	67.4 (3,169)	41.12 (3,169)
	<0.0001	<0.0001	<0.0001	<0.0001
Adjusted *R*^2^	8.5%	5.8%	32.5%	22.5%

* Significant.

## Discussion

This multinational study was conducted ~2 years after the declaration of COVID-19 as a pandemic. The current study represents a large-scale evaluation of the QoL among HCWs in several Arab countries. Our findings pointed to generally poor QoL among HCWs in the Arab world, with better mean scores for general and physical health while the social, psychological, and environmental domains showed the lowest QoL scores. Most of Arab countries suffered from a high burden of COVID-19, with HCWs experiencing such a high burden of the disease due to their frontline position in the fight against the pandemic (25–27). Thus, the assessment of QoL among HCWs during the pandemic can be viewed as a timely and relevant aim considering the high levels of psychological problems and burnout that HCWs have experienced during COVID-19 (28–30). This can result in poor QoL among this group, which subsequently may adversely affect the quality and efficiency of care delivery (13).

### The QoL among HCWs

In this study physicians, pharmacists, and physiotherapists had lower mean QoL scores compared to nurses and health co-workers. However, the difference observed across occupational categories was around 3 points. This result can be related to the nearly equal risk of COVID-19 infection across different categories taking into account their key role in the pandemic control, with minor risk differences. A similar finding was reported by de Paula et al. (31), in a study that involved a total of 95,397 HCWs in Brazil. In the Brazilian study, nurses and health co-workers (physical education, non-health related professions) displayed higher QoL scores compared to physicians with a mean difference of 5 points maximum in QoL score.

Across the four domains of WHOQOL-BREF evaluated in this study, the highest mean score was found in the physical domain 68.0 ± 15.7 (with 40.8% participants having normal domain score), indicating sufficient energy, ability to cope with fatigue, pain, and discomfort, and adequacy of sleep and rest. However, this result was variable across different HCWs' categories and participants' characteristics. Similar observation was reported in a recent study among Malaysian HCWs (13).

In this study, the lowest mean score was observed for the social domain, 53.5 ± 23.0 (26.2% had a good social relationship). Low scores in social domain are likely related to social circle dissatisfaction, workload and long working hours. This involves more negative feelings, and a lower level of self-esteem, which can be connected to the consequences of the ongoing pandemic. Similarly, low mean scores were observed for the environmental 60.3 ± 12.7 (15.4% had normal domain scores) and in the psychological domains 55.9 ± 17.9 (22.3% had normal domain scores), indicating poor satisfaction in a home environment, poor participation in recreation activities, compromised activities of daily living, general law and order situations, less mobility and more discomfort, fatigue, and less work capacity. The highest score observed in this study was for the physical domain, 68.0 ± 15.7 (40.8% had good physical health). This implies better self-care, mobility, and physical activity level, or due to the sample characteristics entailing a working population that is usually healthier than non-working individuals (32). Conversely, Iqbal et al. (33), reported that Pakistani HCWs had higher mean scores for the psychological (68.9 ± 15.5) and social domains (70.3 ± 15.9) compared to physical (65.2 ± 13.0) and environmental domains (65.1 ± 15.2). This indicates good individual relationships, greater social support and satisfactory sexual activities, average financial means, average healthcare facilities, and availability of cheaper but convenient transportation. Of note, all these scores were higher than scores calculated in this survey. This may be due to the difference in the timing of surveys, with the Pakistani study being conducted in December 2020 after less than a year since COVID-19 was announced as a pandemic.

### Determinants of QoL among HCWs

In the current study, the main determinants of the physical domain were male gender, residence in urban settings, the participants' country income-level, higher educational level, income per capita, history of COVID-19, and history of COVID-19 mortality among relatives. The main determinants of the psychological domain were residence in urban settings, the participants' country income level, higher educational level, working sector, income per capita, history of COVID-19, history of previous COVID-19 infection. For the social domain, the main determinants were male gender, the participants' country income level, being single, working sector, being physician, income per capita, receiving COVID-19 vaccine. Lastly, the main determinants of the environmental domain were country of residence, the participants' country income level, income per capita, uptake of COVID-19 vaccination, and having relative who died due to COVID-19.

In the current work, age was not a significant determinant of the physical activity score. A similar finding was reported by Rahman et al. (34). On the other hand, individuals over the age of 45 were 52% less likely to be in good physical health compared to younger participants (35). In fact, 76.4% of the study participants were 40 years and below, which may explain the non-significant effect of age on physical QoL as most of the recruited HCWs were relatively young. Moreover, around four-fifth of the study participants did not report any history of chronic diseases that may hinder their physical activity. The working experience in years was significantly associated with the social and environmental domains of QoL; however, in multivariable analysis this variable was not significant. To the contrary, A previous study speculated that staff beyond the age of 40 years would have more work experience and greater knowledge of the nature of work and working circumstances (9). Being a male was significantly associated with higher physical and social means scores, while being single was associated with a significant decline in the mean score of social domains (β = 24.2). Similarly, the married HCWs in this study had higher mean scores for the social domains and general health (33, 36). Statistically significant differences were observed between HCWs in rural and urban areas in the physical, psychological, and environmental domains of the WHOQOL-BREF. In this study, HCWs working in urban areas displayed higher scores, despite the more demanding and stressed practice environments. We speculate that the higher income in urban areas, the type of work, and experience may cause such an effect. It is worthy to note that income per capita was a significant predictor of all domains of QoL. On the other hand, Iqbal et al. (33), found that HCWs working in rural areas had higher social and environmental domains due to the less stressful lifestyle. Diener et al. (37), found that economic prosperity improves QoL; wealth was associated substantially with 26 of the 32 indicators examined in their study, showing that wealthier countries have better QoL. On the contrary, Tang et al. (38), reported that income did not affect QoL. In our sample, income was affected by the occupation, and being a physician significantly affected the social relationship domain. Similarly, Nordt et al. (39), reported that QoL increased considerably and was judged higher by individuals with any type of work than by persons without a job. Populations with higher education levels frequently promote self-interest and engagement in improving overall health conditions. Furthermore, those with higher education are more likely to change their lifestyle and implement preventative measures, resulting in improved QoL (40). In this study, highly educated HCWs had better social relationships and psychological domains when compared to others.

### COVID-19 impact on the QoL of HCWs

In the current study, the physical and psychological domains were significantly different among participants who experienced a history of COVID-19. This may be due to the negative impact of COVID-19 on sleep pattern and induced anxiety (41). Similarly, in a systematic review of 4,408 patients who tested positive for COVID 19, the QoL has considerably been influenced regardless of the period following hospital discharge. The most affected domain was the physical domain, especially among those who developed severe illness and those who required Intensive Care Unit (ICU) admission (42). Furthermore, Shah et al. (43), found that COVID-19 survivors experienced significant long-term effects on their physical and psychological wellbeing. Their partners' and other family members' lives were also significantly impacted. Hospitalization or quarantine due to diseases or illnesses can effectively reduce the QoL among survivors (39, 44). We found that, except for the environmental domain, the fully vaccinated subjects had a non-significant difference compared to non-vaccinated. Contrary to a large survey study that was conducted in Poland with 1,696 respondents to determine the effect of COVID-19 vaccination on QoL, fully vaccinated individuals had higher QoL scores and lower subjective anxiety about being infected with COVID-19 compared to individuals awaiting vaccination or with an incomplete vaccination regimen (one dose). This finding may indicate the non-confidence in COVID-19 vaccine effectiveness despite receiving vaccination or might be related to the workload assigned to those vaccinated in comparison to those who were not (45, 46). Many countries obliged vaccination among citizens to receive governmental services. This may be one of the main causes to receive the vaccination (47). Death of relatives decreased the mean score of all domains, yet, only the physical and environmental domains were significantly affected in this study. To the contrary, Ham et al. (48), found that the COVID-19 pandemic had no substantial influence on the wellbeing of grieving families in the short term. We speculate that the effect was not apparent as the sample size was relatively small (*n* = 91), which may not be representative of the population. In addition, many of them died from diseases other than COVID-19 like cancers and died at home. These diseases might have made the death of relatives expected.

### Implication of this study

Indeed, it is crucial to ensure that HCWs had an acceptable level of QoL as they form an essential component within the health-system framework. Their wellbeing is required for better patient care and hence safer outcomes. Unfortunately, most studied on Arab HCWs showed low scores of all domains of QoL. This is an alarming finding and it should be further investigated. Policy makers and stakeholders should work to improve QoL of HCWs in order to augment their performance and to enhance job satisfaction. Organizational level interventions targeting work environment in addition to individual psychological support will mitigate the negative impact of COVID-19 disease. Moreover, this would maintain their resilience and guard against burnout.

### Strengths and limitations

To the best of our knowledge, this is the first study that assesses QoL among HCWs in the Arab countries as a multi-national study. The sample size was large, exceeding three thousand participants from countries of different income levels (low-, middle-, and high-income countries). The tool used in data collection is a validated version of the QoL used in three different languages (Arabic, English, and French). This helped to recruit participants of different backgrounds and nationalities. One of the study limitations is the type of study being cross sectional that may create bias and the non-random sampling method that may hinder the generalization of the research outcome. Further comparative studies before and after the pandemic may provide more insights on the effect of the pandemic on QOL. Second, due to the absence of focal points or conflicts and wars, some countries were not represented (Algeria, Djibouti, and Comoros) or underrepresented (Bahrain and Syria). Finally, this survey was conducted online; however, due to the measures implemented to combat SARS-CoV-2 transmission and its variant strains, this data collection method was the most suitable.

## Conclusions

In conclusion, most HCWs in the Arab world have unsatisfactory scores of QoL across different domains (physical, psychological, social, and environmental) except for the general QoL. This may shed light on the importance of improving their QoL to ensure the quality of services provided to the patients. The experience of COVID-19 and its consequences are likely incriminated in lowering the QoL of HCWs, primarily as they represent the key group in combating this pandemic, while vaccination against COVID-19 did not improve the QoL.

## Data availability statement

The original contributions presented in the study are included in the article/Supplementary material, further inquiries can be directed to the corresponding author.

## Ethics statement

The studies involving human participants were reviewed and approved by Alexandria University N 00012098. The patients/participants provided their written informed consent to participate in this study.

## Author contributions

RG conceptualized of the research idea, participated in data collection, statistical analysis, writing manuscript, and respond to reviewers' comments. OA participated in data collection, statistical analysis and manuscript writing manuscript and review. EH co-ordinated the study, reviewed and adapted questionnaire, data collection, and writing and reviewing manuscript. EB reviewed and adapted questionnaire, data collection, data analysis, and writing and reviewing manuscript. SA participated in data collection, manuscript writing manuscript, and review. SM and RA participated in data collection, data analysis, interpreting result, and writing manuscript. IE participated in questionnaire review and adaptation, data collection and writing and editing manuscript. All other co-authors participated in data collection, editing, and final review of the manuscript.

## Conflict of interest

The authors declare that the research was conducted in the absence of any commercial or financial relationships that could be construed as a potential conflict of interest.

## Publisher's note

All claims expressed in this article are solely those of the authors and do not necessarily represent those of their affiliated organizations, or those of the publisher, the editors and the reviewers. Any product that may be evaluated in this article, or claim that may be made by its manufacturer, is not guaranteed or endorsed by the publisher.
